# Psychometric Analysis of a Postulated Set of Evolved Human Motives

**DOI:** 10.3389/fpsyg.2021.680229

**Published:** 2021-07-29

**Authors:** Robert Aunger, Dugald Foster, Val Curtis

**Affiliations:** ^1^Environmental Health Group, Department of Disease Control, London School of Hygiene and Tropical Medicine, London, United Kingdom; ^2^College of Life and Environmental Sciences, University of Exeter, Exeter, United Kingdom

**Keywords:** motive, motivation, evolutionary pscyhology, factor analysis, behavior determination

## Abstract

Many different general systems of human motives have been postulated in the psychological literature. However, as yet, no consensus on which motives should be nominated, nor how many there are, has emerged. Recently, we deduced the existence of a number of motives using a logical argument derived from evolutionary theory; that humans have evolved an independent psychological “engine” to respond to each kind of evolutionary problem set by a dimension of the human niche, or life-way. Here, we confirm the existence of 14 out of 15 of these postulated motives using factor analysis on a web-based sample of 500 respondents from the UK: Lust, Hunger, Fear, Disgust, Attract, Love, Nurture, Hoard, Create, Affiliate, Status, Justice, Curiosity, and Play. The items which loaded most strongly for each factor confirmed the expected core value of each motive. Comfort did not emerge, perhaps because it is more about satisfying specific physiological requirements than a cluster of activities linked semantically by the concept of attaining “comfort.” We believe this analysis can form the foundation of a scale for use in applied psychological work ranging from personality testing to personnel selection to public health program design.

## Introduction

Understanding motivation is key to understanding our own behavior and that of others. However, psychologists have yet to agree as to the existence of separable motives (Barrett, 2017), how many there are, nor on the identity of individual motives. Many different systems of human motives have been postulated in the psychological literature, from its earliest days (James, 1890) to the present (Talevich et al., 2017; Chierchia et al., 2020; Desmet and Fokkinga, 2020; Ko et al., 2020). Scholars have used a wide variety of approaches to identify sets of motivational constructs (sometimes called by names such as ergs, drives, needs or goals). For example, Murray came up with a list of 23 “psychogenic” needs including for water, achievement, seclusion, order, and exposition as part of his theory of personality (Murray, 1938). Maslow famously postulated a hierarchy of 12 needs including affiliation, achievement and security from his experience as a primatologist (Maslow, 1943). Cattel carried out factor analysis on agglomerations of linguistic terms to identify ten motives including affiliation, nurturance and safety (Cattell, 1957). Similarly, Chulef and colleagues, Chierchia and colleages, and Talevich and colleagues used hierarchical clustering to reduce the large number goals, values and needs identified in the history of psychological literature on human motivation to a more manageable list based on semantic similarity judgments (Chulef et al., 2001; Talevich et al., 2017; Chierchia et al., 2020). Reiss and Havercamp reviewed lists generated by previous scholars to derive a set of 16 basic desires including curiosity and citizenship (Reiss and Havercamp, 1998). Some authors picked a single motive which they argued must be central to any explanation of human behavior—such as the need for achievement (McClelland et al., 1953), power (Winter, 1973), or affiliation (Schachter, 1959; Baumeister and Leary, 1995). Hybrid methods have also been used. Desmet and Fokkinga began with Maslow's hierarchical typology of needs, but also included candidates identified by several other studies that satisfied a set of five criteria for only identifying “fundamental” needs. From this list the authors used a variety of techniques, such as collapsing similar needs, to arrive at a consensual list (Desmet and Fokkinga, 2020).

Each of these approaches to the identification of human motives has shortcomings. For example, it is unlikely that English language categories mirror psychological categories precisely, nor that all kinds of motivation are expressed linguistically. Combining and selecting from the work of previous authors without strict criteria lacks rigor and postulating individual or small sets of motives fails to provide a complete picture of the domain of human motivation.

An alternative means of identifying human motives is to define them *a priori* based on some argument from theory. For example, Bugental recently identified a logical suite of social relationship types that must be motivated (Bugental, 2000), while Kenrick and colleagues relied on a combination of functional and evolutionary arguments to update Maslow's hierarchy of needs (Kenrick et al., 2010), producing a set of four motives including safety/security and reciprocity. However, these sets are incomplete because they are restricted to the social realm. Humans, while being the most social species on the planet, must still avoid accidents, and find food and shelter to survive, and so are motivated to achieve physical and biological goals as well as social ones.

To identify a complete and comprehensive set of distinct human motives, we deduced their existence using a logical argument derived from evolutionary theory (Aunger and Curtis, 2013). Our supposition was that the human way of life is associated with the need to satisfy a range of evolutionary goals tied to reproduction and survival. Essentially, each dimension of the human niche requires us to perform behaviors that achieve a specific kind of objective. For example, all species need to reproduce; human do this sexually, which requires individuals to seek out and mate with, partners—a motive we call Lust. However, our offspring are also dependent on long-term care to survive. Providing this care involves a pair-bond. The motive to maintain such a pair-bond we call Love.

These objectives are associated with distinct end-states that produce evolutionary benefits for those that achieve them, such as copulations and surviving offspring in the two cases just discussed. We then noted that the evolutionarily ultimate end-states of reproduction and survival can be achieved more or less directly by a particular behavior. Behavior can benefit the individual directly (in terms of actually reproducing through copulation or aiding somatic survival—as when avoiding predation). But behavior can also result in end-states that are only indirectly related to these ultimate goals, by producing improvements in the individual's physical, biological or social situation, or even more indirectly, by investing in psychological abilities to improve their situation in future. That is, individuals can engage in Creating a more supportive environment by constructing shelter (to protect from potential predation), or planting crops to be eaten later. Alternatively, a person can engage in repeated practice of a hard-to-learn skill such as hunting animal prey (which we call Play), that can benefit one's hunting efficiency subsequently. In this way, 15 kinds of end-states were identified: ranging from consumption of nutritious food, to recognition of status by a social interactant, to investing in knowledge of where fruit trees are blossoming or predators are lying in wait, and so on. We further postulated that there should be a specific kind of “mental engine” to drive the behaviors leading to the achievement of each of these objectives, since each one can involve recognition of different situations as opportunities or threats of a particular kind, as well as identification of the most likely sequence of behaviors necessary to take advantage of the opportunity or avoid the specific kind of threat present. These situations and response sequences can be quite distinct (e.g., a mating opportunity bears little resemblance to attack by a predator, and the behavioral responses are opposite). The need to quickly identify the correct response can lead to psychological specialization in the form of a modular mechanism. We call these psychological mechanisms “motives.” They are: Lust, Hunger, Comfort, Fear, Disgust, Attract, Love, Nurture, Hoard, Create, Affiliate, Status, Justice, Curiosity, and Play (see Table 1 for a brief definition of each). If the motives that we postulate each result in a unique kind of behavioral bias in response to particular kinds of stimulus, we should be able to identify these motives using standard psychometric techniques.

**Table 1:** The evolved human motives.

Lust	Desiring sexual union with another person
Hunger	Desiring to eat food or drink
Comfort	Avoiding bodily discomfort
Fear	Avoiding physical attacks and accidents
Disgust	Avoiding infection
Attract	Seeking to inspire sexual interest
Love	Seeking to maintain a pair-bond
Nurture	Seeking to promote the interests of one's offspring/gene copies
Hoard	Seeking to always have the things needed to be prepared for any situation
Create	Seeking to improve one's physical surroundings
Affiliate	Seeking to behave in ways that make others want you in their group
Status	Seeking esteem and respect from others
Justice	Desiring to punish anyone who does anti-social things
Curiosity	Seeking information about what's going on in the world
Play	Seeking to learn new skills

In this study we therefore employed confirmatory factor analysis to identify factors related to these motives on a web-based sample of individuals from the United Kingdom. We analyse and discuss the results, and conclude by arguing that the (slightly modified) set of motives that we have thus identified should be used generally by psychologists to understand motivated human behavior.

## Materials and Methods

We used confirmatory factor analysis to conduct a psychometric study of human motivation. Factor analysis is a statistical procedure which is used to identify dimensions along which datasets vary systematically. It takes a suite of statements (called “items”) and reduces them to a smaller set of factors that share a common response pattern from a population of respondents. These factors can then be interpreted (Brown, 2014). Psychometric factor analysis takes advantage of the fact that individuals are likely to vary in the weight they attach to achieving different kinds of objectives, even though we expect everyone (within the normal range of psychological functioning) to place some value on all of these outcomes (Thurstone, 1954; DeVellis, 2016). Confirmatory, rather than exploratory, factor analysis, is appropriate when attempting to verify the existence of a pre-defined set of factors.

### Item Generation and Selection

The study authors used the definitions of the evolutionary function of the 15 postulated motives to generate a large number of potential items, which were then subjected to preliminary data collection and analysis. How well each item reflected the central evolutionary function of the motive was discussed, and items eliminated by consensus. An online version of the 120 selected item list was hosted by Webexperiment.com for 6 months, which generated a sample of 147 respondents. In order to broaden the demographic and geographic range of the sample, we advertised the same list via Facebook for a week, accumulating a total of 419 responses (including the original 147). In a first step, several items were removed from analysis due to significant skew. We used Maximum Likelihood analysis with Varimax rotation, as this was an exploratory phase, and restricted solutions to between 11–20 factors. No distinct factors were found for Hoard, Disgust, Comfort or Play, and those for Create and Status were not very clearly reflective of their nature. No significant cross-correlations were found.

For the second round of data collection and analysis, additional items were generated for motives for which there were too few items for reliable estimation and for motives which did not have a factor at all. This resulted in a list of 10 candidate items for each motive (see Table 1; 10 items being commonly used as a reasonable size for a stimulus set to identify a latent variable such as a factor) (Marsh et al., 1998; Brown, 2014). The additional items were produced independently and together in several new brain-storming sessions, and then agreed to a final selection by consensus, based again on how closely the item was seen to reflect the central purpose of each motive. Each item was phrased as a statement, or value attached to a statement (Fishbein and Ajzen, 1975). This might be expressed as a factual consequence of behavior (“I don't have many friends;” “I read more fiction than non-fiction”), a behavioral tendency (e.g., “I wouldn't mind getting close to the edge of a cliff;”), a liking of a certain kind of reward from behavior (“Doing the little things that are needed to make sure a child is safe and secure give me satisfaction”), or experiencing a specific kind of consequence from behavior (“[Seeing] gory images make me feel faint”). Although they do not ask respondents to report about their behavior directly, each is a good indicator of some aspect of behavior. For example, not having many friends is an indicator of not seeking out affiliations. The complete list of 150 items is listed in Appendix.

### Study Transparency

The hypothesis and analytical strategy of this study were pre-registered on the Open Science Framework prior to data collection (pre-registration available from https://osf.io/hr8s6/register/5771ca429ad5a1020de2872e). Statistical scripts (in R) used in data cleaning, manipulation and analysis are available from the authors. The data themselves have been archived at https://ukdataservice.ac.uk.

### Sample Size

Based on the number of model parameters (survey items), the size of expected loadings (where expectations were derived from prior studies of a similar kind), and known model size, we determined on a sample size of 500 (MacCallum et al., 1999). Ultimately our sample consisted of 510 responses due to an extra 10 participants freely provided by the online survey service.

### Ethics

The study protocol was approved by the London School of Hygiene and Tropical Medicine Ethics Committee prior to data collection (internal application number 5734).

### Data Collection

Data were collected by the Qualtrics company (www.Qualtrics.com) using Qualtrics v.10/18. To maximize representativeness, we requested that individuals from their large empanelled population be recruited until age, gender and regional quotas matched proportions from recent UK census data. Respondents were tasked with completing an on-line questionnaire consisting of a study consent form, 150 items (statements), and demographic information. Respondents were paid £4.00 for completing this task. Data were collected in two rounds between September and October 2018. In the first (pilot) round, 50 responses were collected in order to check for data quality issues, and to decide on a cut-off point for survey completion times. No changes to items were made. In the second round, responses from 460 participants were collected, resulting in a total sample of 510 for final analysis. Following standard Qualtrics practice, participants with completion times under two thirds of the median completion time from the pilot round (11 mins) were dropped (for responding too fast) and replaced by an extra participant. Participants that incorrectly answered a random attention check were also replaced.

Each item consisted of a statement to which respondents provided answers using a 5-point Likert scale (“Strongly disagree,” “Somewhat disagree,” “Neither agree nor disagree,” “Somewhat agree,” “Strongly agree”). Items were presented in random order on the website, but in the same order to all respondents.

### Data Analysis

All analyses were conducted using R v3.5.0 (R Core Team, 2018) and RStudio v1.1.447 (RStudio Team, 2016).

#### Exploratory Analyses

In order to assess whether it was reasonable to extract our predicted number of factors (15), we conducted an exploratory factor analysis of the Qualtrics data. This involved using a random subset of the data (*n* = 255) to estimate eigenvalues from a scree plot using the *psych* package in R (Revelle, 2017).

#### Confirmatory Analyses

We then conducted Confirmatory Factor Analysis (CFA) to test whether a theoretically derived model of the covariance among variables provided a good fit to the data. Due to the likelihood that the factors are inter-correlated rather than orthogonal (that is, we expect there to be internal structure to the relationships among the motives), allowing non-orthogonal estimation made more sense. For the CFA analyses we therefore used the *lavaan* package in R (Rosseel, 2012). Our *a priori* factor structure was derived from our pre-registered model which included 15 factors, representing the 15 different postulated motives.

Following common practice, we compared models using both the Root Mean Square Error of Approximation (RMSEA) and the Comparative Fit Index (CFI). Models were considered to provide a good fit to the data if they showed an RMSEA ≤ 0.08 and a CFI ≥ 0.9. After evaluating the model fit, we calculated overall consistency value (α) and cross-correlation indices.

## Results

### Descriptive Statistics

Table 2 shows the characteristics of our sample, which displayed standard demographic characteristics consistent with the British population as a whole.

**Table 2:** Sample characteristics.

	**Variable**	**Value%**
Age group	18–24	11.4
	25–34	17.2
	35–49	25.2
	50–64	23.5
	65+	22.7
Gender	Male	48.8
	Female	51.2
UK Region	North East	4.1
	North West	11.0
	Yorkshire and the Humber	8.3
	East Midlands	7.2
	West Midlands	8.7
	East of England	9.3
	London	13.2
	South East	13.7
	South West	8.5
	Wales	4.8
	Scotland	8.4
	Northern Ireland	2.8

### Exploratory Factor Analysis

**Figure 1:**
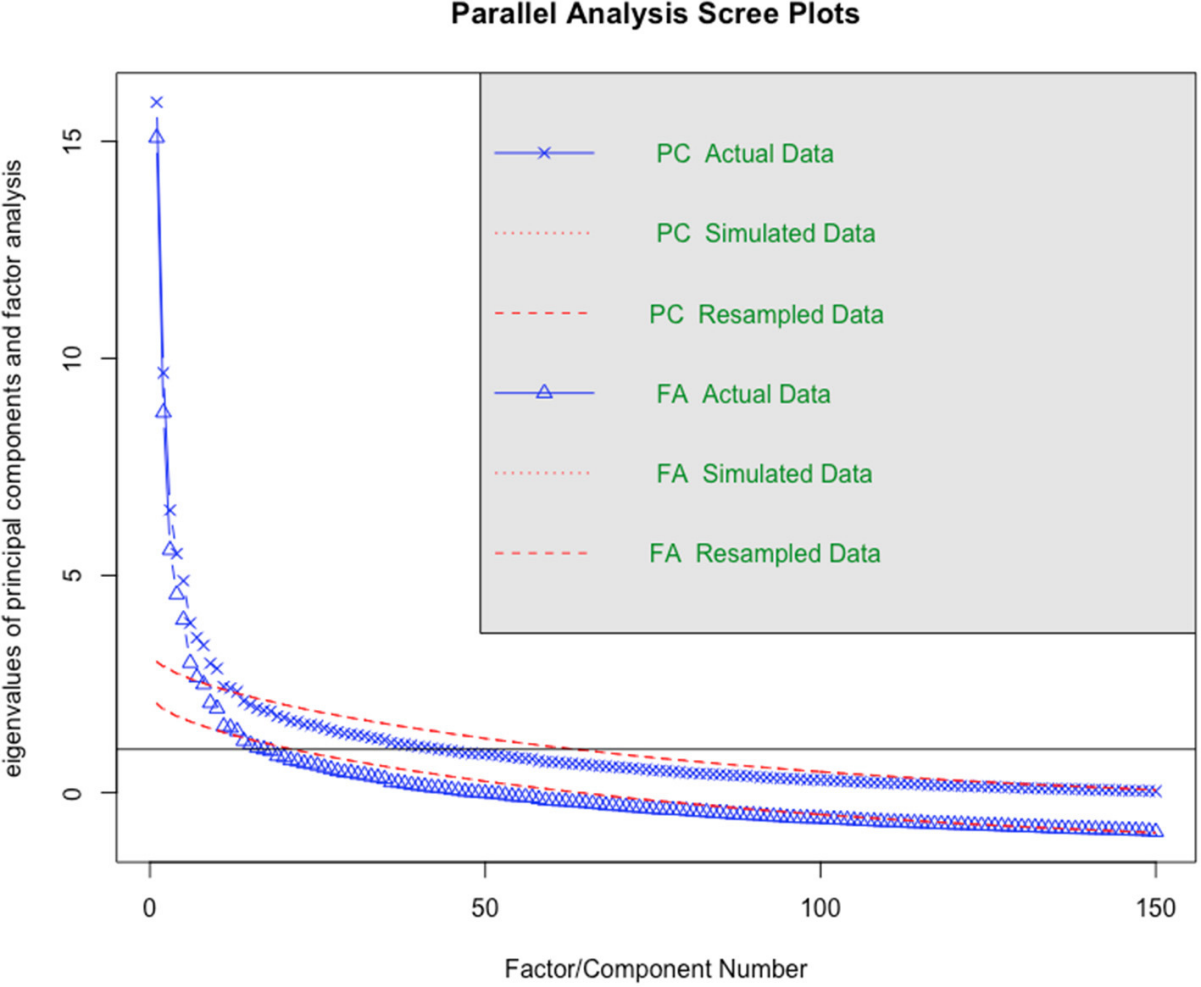
Scree plot analysis.

A scree plot analysis (see Figure 1) showed that we should be able to extract around 13 factors from simulated datasets (based on the point at which the “elbow” in the plot for the simulated and actual FA data occur) (Cattell, 1966).

### Confirmatory Factor Analysis

Next, in line with the study pre-registration protocol, we ran a CFA analysis of the complete 15-factor model on the full sample of 510 respondents (see Table 3, left hand side for results). This analysis showed that all but one factor produced reasonable factor loadings. However, the overall measures of fit (i.e., the CFI and RMSEA values) for this model were not sufficiently robust (0.317 and 0.138, respectively). We therefore decided to investigate further.

**Table 3:** CFA model results.

**15 factor model**					**14 factor model**			
**Item**	**Estimate**	**Std. Err**	***z*-value**	***P*(>|*z*|)**	**Estimate**	**Std. Err**	***z*-value**	***P*(>|*z*|)**
**Lust**								
Q1	0.795	0.020	40.205	0.000	0.760	0.024	31.334	0.000
Q2	−0.739	0.024	−30.687	0.000	**−0.843**	0.026	−32.264	0.000
Q3	0.801	0.019	43.135	0.000	0.733	0.025	29.017	0.000
Q4	−0.790	0.021	−38.302	0.000	**−0.819**	0.024	−34.239	0.000
Q5	−0.606	0.031	−19.364	0.000	−0.587	0.042	−13.940	0.000
Q6	0.440	0.038	11.719	0.000	0.309	0.051	6.016	0.000
Q7	−0.830	0.018	−45.828	0.000	**−0.854**	0.022	−38.455	0.000
Q8	−0.143	0.043	−3.291	0.001	−0.357	0.049	−7.217	0.000
Q9	0.586	0.032	18.492	0.000	0.487	0.043	11.416	0.000
Q10	0.414	0.039	10.594	0.000	0.370	0.048	7.730	0.000
**Hunger**								
Q11	0.753	0.021	36.019	0.000	0.573	0.036	16.003	0.000
Q12	0.734	0.023	32.187	0.000	0.694	0.034	20.459	0.000
Q13	0.580	0.030	19.085	0.000	0.349	0.049	7.084	0.000
Q14	−0.563	0.031	−17.989	0.000	−0.560	0.041	−13.579	0.000
Q15	−0.640	0.026	−24.453	0.000	**−0.737**	0.031	−23.762	0.000
Q16	−0.571	0.030	−19.061	0.000	−0.698	0.031	−22.873	0.000
Q17	−0.685	0.025	−27.552	0.000	**−0.731**	0.033	−22.355	0.000
Q18	−0.512	0.032	−16.112	0.000	−0.559	0.040	−13.906	0.000
Q19	−0.486	0.036	−13.478	0.000	**−0.735**	0.042	−17.712	0.000
Q20	0.784	0.020	39.112	0.000	0.647	0.036	18.048	0.000
**Comfort**								
Q21	0.360	0.047	7.586	0.000				
Q22	−0.057	0.051	−1.125	0.261				
Q23	0.376	0.045	8.293	0.000				
Q24	0.186	0.049	3.790	0.000				
Q25	0.042	0.050	0.844	0.399				
Q26	0.720	0.039	18.689	0.000				
Q27	0.699	0.039	18.065	0.000				
Q28	0.424	0.046	9.188	0.000				
Q29	0.427	0.049	8.685	0.000				
Q30	0.387	0.042	9.156	0.000				
**Fear**								
Q31	0.373	0.048	7.811	0.000	**0.624**	0.051	12.203	0.000
Q32	−0.411	0.044	−9.288	0.000	0.177	0.060	2.969	0.003
Q33	0.475	0.043	11.145	0.000	0.380	0.056	6.766	0.000
Q34	−0.359	0.047	−7.612	0.000	−0.108	0.064	−1.681	0.093
Q35	−0.668	0.039	−17.134	0.000	**−0.490**	0.053	−9.206	0.000
Q36	0.272	0.047	5.741	0.000	0.308	0.057	5.411	0.000
Q37	0.520	0.042	12.426	0.000	**0.592**	0.042	13.979	0.000
Q38	0.536	0.040	13.530	0.000	0.395	0.054	7.285	0.000
Q39	−0.441	0.045	−9.714	0.000	0.082	0.059	1.379	0.168
Q40	−0.339	0.045	−7.501	0.000	0.117	0.063	1.865	0.062
**Disgust**								
Q41	0.594	0.041	14.477	0.000	0.409	0.057	7.137	0.000
Q42	0.484	0.041	11.812	0.000	0.299	0.059	5.050	0.000
Q43	0.609	0.041	14.861	0.000	**0.606**	0.060	10.087	0.000
Q44	0.603	0.037	16.433	0.000	**0.585**	0.051	11.500	0.000
Q45	0.238	0.048	4.972	0.000	0.065	0.067	0.971	0.332
Q46	0.582	0.039	14.865	0.000	0.584	0.049	11.927	0.000
Q47	0.295	0.050	5.918	0.000	0.035	0.072	0.487	0.626
Q48	0.332	0.049	6.746	0.000	0.134	0.067	1.989	0.047
Q49	0.472	0.046	10.297	0.000	0.438	0.065	6.726	0.000
Q50	0.258	0.046	5.595	0.000	**0.656**	0.065	10.132	0.000
**Attract**								
Q51	0.662	0.030	21.742	0.000	0.672	0.031	21.526	0.000
Q52	0.754	0.026	29.114	0.000	**0.732**	0.030	24.346	0.000
Q53	0.630	0.030	21.230	0.000	0.665	0.033	19.959	0.000
Q54	0.557	0.033	16.642	0.000	0.652	0.033	19.947	0.000
Q55	−0.129	0.045	−2.896	0.004	−0.230	0.050	−4.610	0.000
Q56	0.744	0.026	28.661	0.000	**0.700**	0.031	22.492	0.000
Q57	−0.181	0.045	−3.986	0.000	−0.149	0.052	−2.875	0.004
Q58	0.714	0.027	26.519	0.000	0.662	0.031	21.036	0.000
Q59	0.678	0.028	23.819	0.000	**0.683**	0.032	21.656	0.000
Q60	0.588	0.035	16.746	0.000	0.556	0.039	14.096	0.000
**Love**								
Q61	0.560	0.034	16.585	0.000	0.414	0.046	8.929	0.000
Q62	−0.822	0.023	−36.092	0.000	**−0.869**	0.028	−31.098	0.000
Q63	−0.653	0.029	−22.374	0.000	−0.711	0.036	−19.527	0.000
Q64	−0.791	0.022	−36.360	0.000	**−0.721**	0.032	−22.570	0.000
Q65	−0.384	0.040	−9.592	0.000	−0.376	0.052	−7.229	0.000
Q66	0.046	0.045	1.037	0.300	0.005	0.053	0.101	0.919
Q67	−0.619	0.029	−21.293	0.000	−0.696	0.034	−20.780	0.000
Q68	0.283	0.044	6.444	0.000	0.133	0.056	2.366	0.018
Q69	−0.713	0.028	−25.844	0.000	**−0.742**	0.033	−22.525	0.000
Q70	0.687	0.028	24.383	0.000	0.666	0.036	18.650	0.000
**Nurture**								
Q71	0.854	0.017	48.953	0.000	**0.833**	0.023	36.701	0.000
Q72	0.829	0.019	44.164	0.000	**0.783**	0.025	30.948	0.000
Q73	0.870	0.015	57.440	0.000	**0.877**	0.019	47.288	0.000
Q74	0.716	0.026	27.807	0.000	0.726	0.029	25.414	0.000
Q75	−0.547	0.035	−15.541	0.000	−0.315	0.050	−6.347	0.000
Q76	0.801	0.023	35.520	0.000	0.756	0.029	26.023	0.000
Q77	0.661	0.029	22.838	0.000	0.614	0.033	18.597	0.000
Q78	0.572	0.033	17.288	0.000	0.662	0.034	19.441	0.000
Q79	0.584	0.030	19.282	0.000	0.722	0.033	21.623	0.000
Q80	0.366	0.042	8.773	0.000	0.533	0.044	12.141	0.000
**Hoard**								
Q81	0.173	0.055	3.163	0.002	0.037	0.061	0.612	0.541
Q82	0.271	0.050	5.412	0.000	0.137	0.060	2.272	0.023
Q83	0.329	0.047	6.960	0.000	0.194	0.059	3.269	0.001
Q84	0.225	0.051	4.430	0.000	0.155	0.055	2.833	0.005
Q85	0.625	0.039	16.217	0.000	**0.577**	0.043	13.477	0.000
Q86	0.605	0.038	15.977	0.000	**0.540**	0.043	12.461	0.000
Q87	0.243	0.053	4.630	0.000	**0.512**	0.052	9.814	0.000
Q88	0.481	0.045	10.642	0.000	0.181	0.055	3.266	0.001
Q89	0.389	0.045	8.636	0.000	0.479	0.046	10.315	0.000
Q90	0.340	0.046	7.392	0.000	0.402	0.051	7.811	0.000
**Create**								
Q91	0.165	0.045	3.656	0.000	−0.060	0.054	−1.120	0.263
Q92	−0.737	0.029	−25.407	0.000	−0.588	0.038	−15.608	0.000
Q93	0.641	0.034	18.833	0.000	0.386	0.049	7.863	0.000
Q94	−0.606	0.032	−19.142	0.000	**−0.752**	0.029	−25.706	0.000
Q95	−0.412	0.041	−9.967	0.000	−0.272	0.050	−5.478	0.000
Q96	0.600	0.035	17.224	0.000	0.374	0.046	8.140	0.000
Q97	−0.545	0.038	−14.389	0.000	**−0.634**	0.038	−16.753	0.000
Q98	−0.474	0.039	−12.094	0.000	−0.514	0.042	−12.132	0.000
Q99	−0.445	0.043	−10.366	0.000	−0.619	0.040	−15.648	0.000
Q100	−0.649	0.030	−21.597	0.000	**−0.742**	0.030	−24.356	0.000
**Affiliate**								
Q101	0.686	0.028	24.436	0.000	0.433	0.044	9.891	0.000
Q102	0.592	0.033	18.168	0.000	0.302	0.048	6.359	0.000
Q103	−0.751	0.024	−31.151	0.000	**−0.703**	0.031	−22.463	0.000
Q104	−0.661	0.030	−22.038	0.000	**−0.656**	0.036	−18.061	0.000
Q105	0.576	0.035	16.508	0.000	0.513	0.041	12.641	0.000
Q106	−0.486	0.036	−13.500	0.000	**−0.624**	0.038	−16.356	0.000
Q107	0.699	0.027	25.759	0.000	0.603	0.037	16.165	0.000
Q108	−0.056	0.046	−1.206	0.228	−0.433	0.046	−9.452	0.000
Q109	−0.243	0.044	−5.511	0.000	−0.390	0.051	−7.722	0.000
Q110	−0.327	0.044	−7.483	0.000	−0.605	0.041	−14.728	0.000
**Status**								
Q111	0.753	0.027	28.184	0.000	**0.694**	0.030	23.235	0.000
Q112	0.634	0.030	21.045	0.000	0.594	0.032	18.696	0.000
Q113	0.745	0.025	29.873	0.000	**0.714**	0.027	26.801	0.000
Q114	0.466	0.037	12.691	0.000	0.571	0.033	17.499	0.000
Q115	0.443	0.038	11.679	0.000	0.559	0.037	15.215	0.000
Q116	0.563	0.034	16.544	0.000	0.635	0.033	19.268	0.000
Q117	0.565	0.035	16.327	0.000	0.478	0.039	12.270	0.000
Q118	0.759	0.026	29.373	0.000	**0.718**	0.028	26.090	0.000
Q119	−0.085	0.047	−1.809	0.071	−0.127	0.049	−2.563	0.010
Q120	−0.126	0.045	−2.775	0.006	−0.187	0.048	−3.876	0.000
**Justice**								
Q121	0.686	0.032	21.578	0.000	**0.650**	0.034	19.061	0.000
Q122	0.612	0.034	18.066	0.000	**0.636**	0.041	15.472	0.000
Q123	0.511	0.037	13.685	0.000	0.543	0.043	12.673	0.000
Q124	0.306	0.044	6.904	0.000	0.302	0.051	5.937	0.000
Q125	0.488	0.038	12.715	0.000	0.438	0.047	9.340	0.000
Q126	0.348	0.042	8.204	0.000	0.444	0.047	9.523	0.000
Q127	0.619	0.036	17.423	0.000	**0.623**	0.045	13.757	0.000
Q128	0.384	0.040	9.563	0.000	0.332	0.049	6.765	0.000
Q129	0.234	0.050	4.710	0.000	0.325	0.055	5.948	0.000
Q130	0.435	0.043	10.051	0.000	0.306	0.057	5.340	0.000
**Curiosity**								
Q131	0.644	0.035	18.160	0.000	**0.624**	0.041	15.114	0.000
Q132	0.535	0.040	13.372	0.000	0.482	0.042	11.570	0.000
Q133	0.621	0.035	17.734	0.000	**0.668**	0.039	17.072	0.000
Q134	0.674	0.035	19.315	0.000	**0.684**	0.033	20.495	0.000
Q135	0.102	0.049	2.098	0.036	0.068	0.051	1.317	0.188
Q136	−0.140	0.049	−2.878	0.004	0.061	0.053	1.152	0.249
Q137	0.617	0.037	16.564	0.000	0.492	0.042	11.600	0.000
Q138	0.563	0.035	15.956	0.000	0.621	0.034	18.283	0.000
Q139	0.149	0.046	3.228	0.001	0.282	0.047	5.978	0.000
Q140	−0.130	0.048	−2.711	0.007	−0.005	0.052	−0.090	0.928
**Play**								
Q141	0.550	0.037	15.048	0.000	0.592	0.035	17.014	0.000
Q142	0.505	0.038	13.286	0.000	0.394	0.040	9.795	0.000
Q143	0.300	0.045	6.589	0.000	0.258	0.045	5.760	0.000
Q144	0.218	0.051	4.301	0.000	0.264	0.046	5.706	0.000
Q145	0.500	0.039	12.936	0.000	**0.602**	0.032	18.978	0.000
Q146	0.475	0.039	12.221	0.000	0.455	0.041	11.204	0.000
Q147	0.722	0.031	23.256	0.000	**0.718**	0.028	25.591	0.000
Q148	−0.415	0.043	−9.589	0.001	−0.267	0.046	−5.747	0.001
Q149	0.369	0.043	8.550	0.000	0.444	0.040	11.227	0.000
Q150	0.615	0.034	18.188	0.000	**0.599**	0.036	16.728	0.000

*We also validated a more manageable scale (a reduced list of the three highest loading items for each of the 14 identified motives, shown in bold in Table 6), showing that it had similar psychometric qualities to the full questionnaire*.

#### 14-Factor Model

We next ran a CFA with one fewer factor than the full model, as allowed by the study pre-registration, in an attempt to improve the model fit by removing the poorly-fitting factor (“Comfort”). This 14-factor model exhibited much better measures of fit (CFI = 0.736; RMSEA = 0.088; see Table 3, right hand side), especially given the large number of factors included in the estimation process.

We also looked at the correlation matrix between factors to ascertain the relationship structure among factors (see Table 4), and calculated the internal consistency (measured as coefficient alpha) for each factor (see Table 5). Overall consistency values (α) for the 10 items per factor range from 0.55 (considered to be relatively low reliability) to 0.86 (reasonably good reliability). Internal consistency is relatively low for Comfort (which didn't actually produce an interpretable factor), Hoard and Curiosity.

**Table 4:** Factor cross-correlations.

		**1**	**2**	**3**	**4**	**5**	**6**	**7**	**8**	**9**	**10**	**11**	**12**	**13**
1	Lust													
2	Hunger	0.201												
3	Fear	−0.256	−0.198											
4	Disgust	0.032	−0.161	0.074										
5	Attract	−0.378	−0.253	0.551	0.290									
6	Love	0.418	0.321	−0.146	−0.269	−0.121								
7	Nurture	−0.061	−0.208	0.261	0.379	0.098	−0.392							
8	Hoard	−0.173	−0.351	0.404	0.208	0.563	−0.326	0.244						
9	Create	0.280	0.179	−0.583	−0.097	−0.422	0.379	−0.359	−0.564					
10	Affiliate	0.218	0.333	−0.394	−0.267	−0.590	0.158	−0.362	−0.295	0.282				
11	Status	−0.182	−0.381	0.483	0.414	0.818	−0.273	0.307	0.617	−0.493	−0.728			
12	Justice	−0.299	−0.245	0.465	0.229	0.198	−0.402	0.458	0.415	−0.579	−0.220	0.330		
13	Curiosity	−0.291	−0.339	0.523	−0.004	0.237	−0.369	0.239	0.561	−0.776	−0.222	0.349	0.714	
14	Play	−0.430	−0.348	0.664	0.139	0.590	−0.370	0.380	0.643	−0.796	−0.530	0.670	0.601	0.870

*N = 510; all |r| > 0.14 are significant at p < 0.01*.

**Table 5:** Internal consistency values by factor.

**Motive**	**α**
Lust	0.82
Hunger	0.83
Comfort	0.55
Fear	0.63
Disgust	0.67
Attract	0.78
Love	0.76
Nurture	0.86
Hoard	0.55
Create	0.74
Affiliate	0.75
Status	0.74
Justice	0.63
Curiosity	0.58
Play	0.64

Finally, we took the three top-loading items per factor and re-ran the CFA analysis on this reduced questionnaire (see Table 6 for a listing of the relevant items, together with the proportion of the sample that agreed with the item statement in the original 10-item analysis). As expected, this analysis showed better measures of fit than the full questionnaire (CFI = 0.866; RMSEA = 0.063).

**Table 6:** Reduced Human Motives Scale^*^.

**Motive**	**Q**	**Item**	**Percent agree/strongly agree**
Lust	Q2	I like to experiment with different sexual positions	50
	Q4	The sheer pleasure of sex is one of life's great rewards	61
	Q7	I hope I'll still be having sex regularly when I get old	61
Hunger	Q15	I really enjoy every bite of what I eat	63
	Q17	Throughout the day, I am always looking forward to the next meal	42
	Q19	I enjoy shopping for food	59
Fear	Q31	I could easily stand up to someone if they threatened me	58
	Q35	I would never go skydiving	63
	Q37	I enjoy going on roller coasters	36
Disgust	Q43	I would be disgusted to find mold on some food I was eating	75
	Q44	Smelling milk that has gone off makes me nauseous	58
	Q50	I always keep my kitchen free from any germs	61
Attract	Q52	I like to dress provocatively	13
	Q56	My friends would say I'm a flirt	23
	Q59	I like to hang out where I might meet desirable partners	14
Love	Q62	I am happiest when I am with a person I love	74
	Q64	I'd rather spend time with my partner than do anything else	57
	Q69	Finding your ideal life partner is the best thing that can happen to you	69
Nurture	Q71	The smile of a child is one of the most beautiful things on the planet	73
	Q72	Being a parent is the most important role one can play in life	68
	Q73	Doing the little things that are needed to make sure a child is safe and secure give me satisfaction	75
Hoard	Q85	I always like to keep plenty of spare items around just in case I need them	62
	Q86	I feel secure when I'm surrounded by stuff that might come in handy	60
	Q87	I'm always buying things that I don't really need	28
Create	Q94	I constantly make small improvements to the things I own	49
	Q97	I would like to build my own house	52
	Q100	I like coming up with new inventions	34
Affiliate	Q103	I spend a lot of time keeping in contact with my friends	37
	Q104	I can't say I know a lot of people	43
	Q106	I prefer to work in a team	37
Status	Q111	Much of what I do is designed to improve my social position	14
	Q113	Holding a well-respected position in society is important to me	26
	Q118	I enjoy showing off things that tell people I'm important	20
Justice	Q121	I would scold anyone who was inconsiderate to others	56
	Q122	I get angry when I see someone take advantage of others	85
	Q127	I am not afraid to stand up for the right thing	79
Curiosity	Q131	It would be a great thrill to discover something no one has ever known before	76
	Q133	I get a lot of pleasure from discovering how things work	65
	Q134	I am fascinated by going to places I haven't visited before	76
Play	Q145	I've always enjoyed play acting	25
	Q147	I love to learn new skills	72
	Q150	I enjoy contemplating new ideas	74

* *Composed of the 3 top-loading items from the original 14-motive factor analysis (see Table 3); values in the final column come from the original, not reduced factor analysis*.

## Discussion

In previous work, we postulated that 15 different motives evolved to bias human behavior toward achieving goals that helped our ancestors to survive and reproduce in our ancestral niche. Here, we sought to test our theoretical predictions empirically by exploring whether our candidate motives can be identified through dimension-reducing (psychometric) techniques. The confirmatory factor analysis suggested that 14 of our 15 hypothesized human motives are dissociable and discreet. Measures of fit (CFI, RMSEA, internal consistency) were acceptable or good.

The fifteenth motive, Comfort, could not be identified as a robust factor using this dataset. This could be because our items simply did not correctly identify the “core” issue associated with this motive (the items which loaded heavily on this factor concerned being lazy—having a lie in, staying in dressing gown all day—rather than sensitivity to pain, hot/cold, touch or loud noises). However, we think it more likely that this hypothesized motive may not be a unitary construct but, in fact, represent a variety of primitive and reflexive responses to physiological stimuli such as light, heat, acidity, wetness or pain. The fact that such perceptions require the use of different senses may mean there is no unified psychological mechanism to be picked up by a factor analysis.

Below we reflect on what the results of the analysis can tell us about each of the other postulated motives, with special reference to the top three loading items, as these figure in the reduced 42-item scale we hope others will take forward (see Table 6). We begin by outlining the motives related to somatic needs.

*Hunger:* Most questions about the hunger motive had high factor loadings and high degrees of agreement, with the exception of “I can go without eating for ages and not think about it.” The three highest loading items concerned the enjoyment of eating, shopping for and anticipation of meals, though caring about food, setting aside time to eat and the pleasure of eating also had high factor loadings. From an evolutionary perspective it seems uncontroversial to suggest that the hunger motive works to drive behavior that provides the immediate, or anticipated, rewards of eating. Food seeking drives (sometimes called instincts or needs) were common in earlier motive schemas—e.g., (James, 1890) and (Maslow, 1943)—but tends not to feature in more recent ones.

*Fear:* Only two fear-related questions had high factor loadings; these were dislike of roller coasters and being able to stand up to a threat from someone else. Unwillingness to go skydiving had moderate factor loadings. There were minor gender differences in the high loading factors and younger people scored more highly on fear (with the exception of the roller-coaster item). Most of the other questions related to imaginary events or non-specific threats that many people may not have actually experienced, such as encounters with predators. It seems that the higher loading questions may concern the unpleasant nature (negative reward) of fearful events that have actually been experienced. Fear or safety and security feature in most motives schemas (Aunger and Curtis, 2013).

*Disgust:* The three highest factor loading items concerned food: “I would be disgusted to find mold on some food I was eating,” “Smelling milk that has gone off makes me nauseous,” and “I always keep my kitchen free from any germs”. The fourth food-related item (“I would not eat any food that had passed its sell-by date”) also loaded highly. Though there was strong agreement about not sharing toothbrushes or not cleaning someone's infected wound, these items had lower factor loadings. Again, it seems that the most strongly loading items on this factor concerned events where participants were likely to have had direct experience of unpleasant effects of contact with disgusting stimuli, potentially being made nauseous or sick in connection with foodstuffs. This is consistent with the well-known “Garcia effect” (Garcia and Koelling, 1966), which accounts for strong food aversions based on bad experiences with food.

A set of needs are linked to the need for mortal individuals to reproduce themselves. In humans, this includes the need to solve problems associated with sexual reproduction and dependent offspring.

*Lust:* 61% of people agreed or strongly agreed that the sheer pleasure of sex is one of life's great rewards; 61% also agreed or strongly agreed that they hoped that they would still be having sex when they got old. These questions had the highest factor loadings, alongside liking to experiment with sexual positions. The questions with lower factor loadings were less directly concerned with the pleasures of sex and more about potentially socially tabooed activities such as one-night stands, early loss of virginity and use of pornography. The core of the lust motive seems thus to be most closely concerned with the directly rewarding nature of sexual activity. This is consistent with evolution having designed the lust motive to drive behavior that maximizes the pleasure derived from this crucial behavior.

*Attract:* Seven out of 10 factors loaded highly (LF > 0.6), suggesting that the analysis has captured an important dissociable psychological factor. The three highest loading questions were: “I like to dress provocatively,” “My friends would say I'm a flirt” and “I like to hang out where I might meet desirable partners,” which have close links to actually finding of a sexual partner. Other high loading items concerned more remote solutions; getting an operation to enhance one's appearance, dieting and exercise, or learning about mating strategies through self-education. Taken together, these items mention a wide range of tactics for attracting the attention and interest of potential partners. Few researchers have proposed attract as a separate motive from love and lust, though Chulef et al. suggest physical appearance is a goal (Chulef et al., 2001). But we believe this result indicates that there is an intermediate goal between immediate satisfaction of sexual cravings (Lust) and long-term pair-bonding (Love).

*Love*: The three highest loading items for the postulated “Love” factor concerned the pleasures of having a life partner (“I am happiest when I am with a person I love,” “I'd rather spend time with my partner than do anything else,” and “Finding your ideal life partner is the best thing that can happen to you”). Three other questions concerning valuing and investing in a partner also loaded highly. Less central were issues concerning dependability and cheating. Again, the core of this motive seems to concern the rewarding aspects of being in a loving relationship. In human evolutionary history, with highly dependent offspring, reproduction tended to be more successful with two parents, so a strong motive to invest in forming and maintaining a pair bond over a long period would have been adaptive (Rotkirch, 2018). Since the high loading items cover willingness to sacrifice for, the central importance of the pair-bond, and a variety of rewards from being in, and maintaining, such a relationship, this factor should adequately represent all the aspects of this important motivation.

*Nurture:* Uniquely, almost all of the items loaded highly on this factor. though the top three were “Doing the little things that are needed to make sure a child is safe and secure give me satisfaction,” “The smile of a child is one of the most beautiful things on the planet,” and “Being a parent is the most important role one can play in life.” Others concerned a willingness to defend a child under threat, despite great potential cost, and a willingness to do alloparenting just for pleasure. Questions that loaded poorly concerned caring for other relatives and the importance of a career versus having children. The results suggest that nurture is one of the most evolutionarily important motives, given that it should be tightly correlated with reproductive success, and that, given the amount of sustained investment that is required to rear a child, the rewards of nurturance must be correspondingly high. What might be missing is expression of the desire to see a child successfully reared to a (high status) adulthood, as the ultimate reward of good nurturing.

There is also a suite of motives related to human social life.

*Affiliate:* We proposed that those ancestors with a strong desire for gaining social acceptance would have had an adaptive advantage in the highly social human niche. The highest loading factors (which loaded negatively) were: “I spend a lot of time keeping in contact with my friends,” “I can't say I know a lot of people,” and “I prefer to work in a team,” which present a somewhat heterogeneous set of indicators of the core value, which suggest we did not identify the core value in this case. On reflection, we did not include any items that concerned the immediate rewards of social behavior (e.g., “I'm happy when I'm with a close friend”), which we suspect might have worked better at identifying the unique qualities of this motive, nor did we identify directly the benefits of working together, collaboratively, which should be central to the appeal of this motive. The items were instead mostly about feelings associated with being in groups or in some cases difficulties that might be associated with trying to maintain relationships.

*Status:* Our proposal from theory was that ancestors who found behaviors related to improving their social position rewarding would have been likely to have enhanced success in securing access to crucial resources. There were seven items with a factor loading above 0.5. These included “I enjoy showing off things that tell people I'm important,” “Holding a well-respected position in society is important to me,” and “Much of what I do is designed to improve my social position”. Items that loaded poorly on this factor concerned being competitive and being in charge. Whilst most of the questions clearly did load together, we provided few items about the rewards of being deferred to or socially recognized (e.g., “It's nice to be admired,” “I'm happy to be complemented when I've done good work”) which may have been more central to this motive. One question, that loaded strongly, comes close, however, by saying “People in my social group look up to me”.

***Justice:****T*here was strong agreement in our data concerning morally-related questions, and clear support in the pattern of responses for our central hypothesis that the Justice motive promotes third party punishment. This is the central mechanism underlying morality in evolutionary models and the consequent ability it confers to cooperate on a large scale, which uniquely characterizes human sociality (Fehr and Fischbacher, 2004; Jordan et al., 2016). The top three loading items were “I would scold anyone who was inconsiderate to others,” “I get angry when I see someone take advantage of others” and “I am not afraid to stand up for the right thing”. Lower loading items concerned attitudes to politics, criminality and direct revenge (“an eye for an eye”).

Other motives concern goals that improve an individual's situation with respect to the physical or biological environment.

*Hoard:* The items that loaded highest on this factor were “I always like to keep plenty of spare items around just in case I need them,” “I feel secure when I'm surrounded by stuff that might come in handy,” and “I'm always buying things that I don't really need”. The highest loading question corresponds closely the hypothesized purpose of the motive in driving behavior that ensured that resources were available for times of scarcity. The top two items refer to the immediate rewards of owning “stuff,” whilst the lower loading items are more distal or abstract (e.g., saving up for the future). Recent motives schemas tend not refer to “hoard” as a motive, though Nohria et al. suggested possession of resources to be a drive (Nohria et al., 2001). Starch and McDougall suggested similar constructs (McDougall, 1908; Starch, 1923).

*Create:* We hypothesized that ancestors who found constructing things such as tools and housing rewarding would have improved their niches, thus putting themselves and their families into a relatively good position for survival and reproduction. The highest loading factors were consistent with this supposition. For example, “I constantly make small improvements to the things I own,” “I like coming up with new inventions,” and “I would like to build my own house” loaded most strongly. There was also strong agreement and high loading for the item concerning the appreciation of good workmanship. Low loading items concerned tidying and watching plants grow. Again, the core of this factor seems to revolve around the pleasurable rewards of constructive behavior. Few recent schemas except Chuleff include constructs related to “create,” though Starch (Starch, 1923), Murray (Murray, 1938) and Maslow (Maslow, 1943) propose needs for aesthetics, beauty and order, which might be seen as an evolved appreciation for highly constructed environments.

Finally, a couple of motives describe how individuals can improve their own mental representations of the world around them or develop skills that enable them to better achieve the goals related to other motives.

*Curiosity:* If Curiosity is essentially about updating one's mental map of the world and storing knowledge about where opportunities and threats lie, as we postulate (Aunger and Curtis, 2013), then it makes sense that the top scoring three items on this factor concerned the direct pleasure of finding things out. These items were: “I am fascinated by going to places I haven't visited before,” “I get a lot of pleasure from discovering how things work,” and “It would be a great thrill to discover something no one has ever known before.” Closely linked, and weighted, was the claim that “I am interested in everything,” which is a somewhat more vague, and less generously rewarded, statement of the same tendency. Again, central to the curiosity motive seems to relate to directly experienced, pleasurable rewards, rather than meeting abstract and distal objectives (studying the genetics of flies, reading fiction or non-fiction).

*Play:* Items concerning the pleasurable rewards of experimental play behavior loaded most highly on this factor. “I love to learn new skills,” “I've always enjoyed play acting,” and “I enjoy contemplating new ideas.” The importance of having fun also loaded highly. The lower loading items concerned losing oneself in reading, sport as a major part of life and playing pranks, which appear to be more distal or abstract aspects of the play motive. There was no difference in the items by gender and little by age, though the play-acting item was agreed to by more younger people. The findings support the notion that the immediate “fun” rewards of experimental, skill-building activity reinforce playful behavior, which would have been adaptive for humans learning to live in their ancestral niches.

### Relationships Between Motives

We can also look at pair-wise relationships between factors by estimating their inter-correlation (see Table 4). Nearly all inter-correlations between the factors are statistically significant, presumaby due to the large sample size; the exceptions being Lust and Disgust (*p* = 0.5), Lust and Nurture (*p* = 0.2), Fear and Disgust (*p* = 0.2) and Disgust and Curiosity (*p* = 0.9). It is obviously interesting to note that Lust does not “mix” with Disgust or Nurture (confusing Lust with either of these can certainly be counter-productive), while Disgust and Curiosity differ at a fundamental level in behavioral terms (one being avoidant, the other involving approach).

We can also look at those correlations which are absolutely large (i.e., |*r*| > 0.6) for indicators of interesting relationships. Play has the highest average correlation with other motives, and relationships with six others at values >0.6: Fear, Hoard, Create, Status, Justice, and Curiosity. The correlation with Create is negative, indicating a difference between practicing a skill and actually producing something. You also can't Play safely unless you are (at least somewhat) Fearful, and can't Hoard the resources needed to engage in practice, while Curiosity can help motivate Playful behavior. The significant relationship with Justice is interesting, suggesting that a concern with fairness can be associated with learning social skills through Play.

On the other hand, Status and Affiliation appear to be opposites (due to a strong negative correlation): aggressively pursuing higher status within a group can apparently work against efforts to be a “good citizen” or member of that group. Curiosity is also inconsistent with a desire to Create a better environment, perhaps because exploration distracts from the focus needed to make something here and now.

### Comparison With Other Schemas

Many alternative motive schemas have been published throughout the history of psychology. Whilst these approaches have produced many similar candidate motives [indeed, those we have defined have been among the more popular ones throughout the history of study on this topic (Aunger and Curtis, 2013)], they also demonstrate considerable disagreement. Part of this lack of agreement concerns what can and cannot be classed as a motive in the first place. We have argued that motives should be seen as a set of evolved mechanisms that achieve goals over the relatively short term through action sequences guided by dopaminergic responses (Aunger and Curtis, 2015). This distinguishes motives from more ancient automated reflexes, and from more recently evolved cognitively planned objectives which require the involvement of consciousness and foreword thinking, as means of controlling the production of behavior. Hence pain avoidance, for example, is ancient and reflexive, whilst “autonomy” (Deci and Ryan, 2000) and “self-actualisation” (Maslow, 1943) are more recently evolved consciously elaborated objectives. As a consequence, we would not class them as motives. Our data support this notion, showing that more abstract and distal objectives do not load so closely to the immediately “rewarding core” of each motive.

Three previous efforts used evolutionary logic to produce lists of human motives, as we did: (Schwartz, 1992; Bernard et al., 2005; Kenrick et al., 2010). Schwartz is by far the most widely used of these schemes. He used a similar logic to ours in his original study of “universal human values” (Schwartz, 1992). He first argued that human values arise because individuals are biological organisms, engage in coordinated social interaction, and that their groups have survival and welfare needs. From these “universal human requirements,” he further deduced eight “motivational types” (prosocial, restrictive conformity, enjoyment, achievement, maturity, self-direction, security, and power), to which he suggested adding tradition, stimulation and spirituality (the last of which was not empirically supported). He then further argued that these ten values could be organized under four higher-level categories, and also arranged in a circumflex, based on possible pairings to achieve these higher goals (which suggested which values a particular value had on either side of itself).

Unfortunately, the results of these three efforts were considerably different from each other, and from ours. Table 7 compares these lists. While there is some overlap, a number of discrepancies also arise between the three typologies. These discrepancies can be largely accounted for by the different starting-points of the authors. Schwartz's intention was to develop a list of values that could be used to compare cultures and behavioral orientations across the world. Kenrick et al. began with a desire to update Maslow's hierarchy of human needs, and Bernard et al. with an *a priori* claim that human motives relate to five ever-expanding realms: “(a) the self-protection domain of the single system; (b) the mating domain of the dyadic system; (c) the relationship maintenance and parental care domain of the small, kin system; (d) the coalition domain of the large, nonkin system; and (e) the “memetic” domain of the large, symbolic, cultural system.” As a consequence, Bernard et al. tend to include more cultural (“memetic”) motives, while Kenrick et al. leave out the motives to improve mental abilities (Curiosity, Play), as well as Justice and Lust, because these don't appear in Maslow's triangle. Schwartz's orientation toward values rather than motives *per se* means that his list contains generic constructs such as “stimulation” and “achievement,” which characterize any goal-oriented activity, but simultaneously lacks specific, basic needs such as sex or love.

**Table 7:** Four evolutionary motive typologies.

**Schwartz, 1992**	**Bernard et al., 2005**	**Kenrick et al., 2010**	**Aunger and Curtis, 2013**
–	Sex	–	Lust
–	–	Immediate physiological needs: liquids, nutrients	Hunger
Hedonism	–	Immediate physiological needs: Overheating	Comfort
Security	Safety	Self-protection	Fear
–	Health	Immediate physiological needs: Disease avoidance	Disgust
–	Appearance	Mate acquisition	Attract
–	Affection	Mate retention	Love
–		Parenting	Nurture
Conformity	Altruism	Affiliation	Affiliation
Power	Material	Esteem/Status	Status
Benevolence	Conscience	–	Justice
–	–	–	Hoard
–	–	–	Create
Self-direction	Curiosity	–	Curiosity
–	Play/Mental	–	Play
–	Aggression	–	–
–	Physical	–	–
–	Meaning	–	–
Tradition	Legacy	–	–
Stimulation	–	–	–
Universalism	–	–	–
Achievement	–	–	–

Our own starting point was to provide an account of the universal, fundamental goals any individual should exhibit—that is, we began our investigation with the desire to identify the means by which humans would need to survive and reproduce, given the features of the human niche (Aunger and Curtis, 2013). This means our list covers much of the ground of the others, including social needs, but not the needs of groups, considered as agents independent of the individuals within them (e.g., group survival is not considered a separate need, as it was by Schwartz). We believe this is a strong foundation on which to build such an important claim about human nature, because evolutionary theory is so well-supported, as the intellectual foundation of the discipline of biology, and by implication psychology, given the fruitful and robust application of evolutionary thinking to psychology already over the past 50 years or so. Certainly, deducing the set of human motives from straightforward theoretical principles should be preferable to inducing them from some select set of data (as in a linguistic corpus) or group of previous studies, as others have done.

Of course, the evolutionary orientation does not distinguish this study from the others just mentioned. Neither does the fact that we use psychometric techniques to validate our list (All of these others have done the same: Schwartz and Boehnke, 2004; Bernard and Lac, 2014; Neel et al., 2016). Using a dimension-reduction statistical technique like factor analysis can produce outcomes that are interpretable from a wide range of starting points. Rather, what we believe we have accomplished here is to have produced empirical support for the existence of a particular set of motives—a very specific choice from among the wide variety of previously postulated motives—that were chosen on the basis of their consistency with a single, theoretically strong proposition that is more general yet parsimonious than the foundations of these other studies, based as it is simply on the claim that human motivation has evolved to solve the problems set by the dimensions of the human niche.

### Limitations

This study was restricted to people living in Great Britain. Obviously, it is desirable when making claims about the universal nature of human motivation to base that argument on evidence that is less WEIRD (i.e., from a Western, Educated, Industrialized, Rich, and Democratic population) (Henrich et al., 2010). Replication with a multi-cultural sample would therefore be desirable. The limited sample size might also have constrained the ability of factor analysis to strongly identify 15 different factors, suggesting that a larger sample might identify the Comfort motive effectively (also suggested by the scree plot analysis). This possibility should also be tested.

### Conclusion

In this paper, we report the results from a study designed to validate the existence of a theoretically-derived set of motives using confirmatory factor analysis. This effort was largely successful, in that 14 of the postulated 15 motives could be rigorously identified. That is, we find statistical support for the notion that there are (at least) 14 simultaneously dissociable human motives which drive behavior that would have been adaptive in our ancestral past. Lust, Attraction, Love and Nurture drive behaviors that are directly associated with reproduction. Hunger, Fear and Disgust fuel and protect the body. Create and Hoard lead individuals to improve their physical niche, whilst Affiliation, Status and Justice lead individuals to improve their social niche. Curiosity and Play motivate individuals to improve their own knowledge and skills, making them better able to profit from their physical and social niche.

Further, each identified motive had much the same meaning as originally hypothesized from theory. That is, each should direct behaviors for achieving exactly the sorts of functions that we expected. For example, Love concerns staying in close proximity to, and spending time with, the pair-bonded individual, while Hoard is about ensuring one is surrounded by all the materials required to survive and prosper in emergency situations.

Our results shed additional light on the operations of these motives. For motives where we provided items that were immediately hedonically rewarding (or punishing) these tended to form the core of the motive. This corresponds closely to a neuroscientific conception of motivation where the experience of past rewards leads to anticipatory motivational value for objects and events generated through the dopamine system (Reeve and Lee, 2019), and hence increased likelihood of repeating that behavior (i.e., reinforcement learning). We cannot say that this is true for all of the motives in this schema because we did not generate hedonically rewarding items for each motive for test. Future work should remedy this omission. However, the pattern is seen strongly in our data. It therefore seems likely that this insight can be used to reliably distinguish nominations of motives: if they aren't accompanied by relatively short-term accomplishment of states through goal-directed behavior that are associated with rewards, then they aren't likely to qualify as motives. Hence, securing a cultural legacy (as suggested by Bernard et al. as a motive) becomes unlikely, due to its requirement for long-term accomplishments, and is more appropriately thought of as a life-plan, while achieving a physiological state of comfort may be pursued so quickly and automatically that it is properly conceived as a reflex. This could be why one of our postulated motives, Comfort, did not emerge from the data. We suggest this is because the goals associated with being “comfortable” are more about satisfying specific physiological requirements than a cluster of activities linked semantically by the concept of attaining “comfort”.

We see the advance made here as consisting in the combination of a rigorous empirical demonstration of the existence of these factors as independent psychological constructs, where the factors represent a set of motives that have been deduced from a single proposition of great generality, which is itself derived from strong theory. This study has demonstrated that 14 motives are mentally constructed in a similar way by a diverse set of people from the UK. It does not prove that that these motives have an influence on behavior, or that they are the only motives which exist (as there might be others in addition to those identified here). However, this set was derived from a strong theoretical basis, and is in many senses common-sensical, as many of these motives have been put forward in previous studies, and are consistent with everyday interpretations of what is important in life. This study simply adds luster to the possibility that a relatively small set of motives can explain much of human behavior.

Having established the existence of these motives, we next examined their inter-relationships, showing that, in terms of instigating behavior, a number of motives appear to be mutually reinforcing (e.g., Status and Attractiveness; Play and Curiosity), while others are generally antagonistic (e.g., Status and Affiliation). Future work should investigate motives' relationships with other sorts of evidence, following the example of recent work in this area linking motive aspects (such as approach/avoid tendencies) and brain area activation (Schultheiss et al., 2021) or examining the perceived real-world importance of different types of motives (Ko et al., 2020). We also validated a more manageable scale (a reduced list of the three highest loading items for each of the 14 identified motives, shown in bold in Table 6), showing that it had similar psychometric qualities to the full questionnaire. We hope that this reduced scale can be used in applied psychological work ranging from personality testing to personnel selection to market testing and public health program design.

## Code Availability

R code for the factor analysis is available from the authors.

## Data Availability Statement

The datasets presented in this study can be found in online repositories. The names of the repository/repositories and accession number(s) can be found below: The data has been deposited at the UK Data Service (https://ukdataservice.ac.uk).

## Ethics Statement

The studies involving human participants were reviewed and approved by the London School of Hygiene and Tropical Medicine Ethics Committee prior to data collection (internal application number 5734). The patients/participants provided their written informed consent to participate in this study.

## Author Contributions

RA and VC conceived of the study. RA led writing of the paper, VC contributed to the writing. DF conducted all statistical analyses and contributed to the description of the data collection and analysis processes. All authors contributed to the article and approved the submitted version.

## Conflict of Interest

The authors declare that the research was conducted in the absence of any commercial or financial relationships that could be construed as a potential conflict of interest.

## Publisher's Note

All claims expressed in this article are solely those of the authors and do not necessarily represent those of their affiliated organizations, or those of the publisher, the editors and the reviewers. Any product that may be evaluated in this article, or claim that may be made by its manufacturer, is not guaranteed or endorsed by the publisher.
