# Supplementary material for: Psychometric Analysis of a Postulated Set of Evolved Human Motives
Source: Front Psychol. 2021 Jul 29;12:680229. doi: 10.3389/fpsyg.2021.680229 (PMC8358141; doi:10.3389/fpsyg.2021.680229)
Supplement: Supplementary file 1 [file Data_Sheet_1.docx]

# Appendix 1: Item List

| **#** | **Questions** |
| --- | --- |
|  | LUST |
| Q1 | Sex is not an important part of my life |
| Q2 | I like to experiment with different sexual positions |
| Q3 | It doesn’t bother me if I go for months without sex |
| Q4 | The sheer pleasure of sex is one of life's great rewards |
| Q5 | I would be happy to have sex outdoors |
| Q6 | I rarely look at pornography |
| Q7 | I hope I'll still be having sex regularly when I get old |
| Q8 | I lost my virginity before most of my friends |
| Q9 | It is difficult to sexually arouse me |
| Q10 | I would never have a "one-night stand" |
|  | HUNGER |
| Q11 | Eating is less important to me than it seems to be for most people |
| Q12 | I don’t really care much about food and drink |
| Q13 | I can go for ages without eating and not even think about it |
| Q14 | When I am hungry I can't think of anything but eating |
| Q15 | I really enjoy every bite of what I eat |
| Q16 | I make sure to set aside some time every day to enjoy a good meal |
| Q17 | Throughout the day, I am always looking forward to the next meal |
| Q18 | I am a greedy person when it comes to food |
| Q19 | I enjoy shopping for food |
| Q20 | I don't get much pleasure from eating |
|  | COMFORT |
| Q21 | I like to pamper myself |
| Q22 | I can tolerate severe cold/heat |
| Q23 | I am a very ticklish person |
| Q24 | I can't stand loud noises |
| Q25 | I could hold up under torture if I had to |
| Q26 | If I could I would spend all day in a cosy dressing gown |
| Q27 | If I'm not meant to be anywhere I'll have a lie in |
| Q28 | If I have a cold I try to take the day off work |
| Q29 | I go to my doctor for minor ailments |
| Q30 | I look forward to going to bed at night |
|  | FEAR |
| Q31 | I could easily stand up to someone if they threatened me |
| Q32 | Just being near large animals scares me |
| Q33 | I wouldn’t mind getting close to the edge of a cliff |
| Q34 | I can't bear watching horror films |
| Q35 | I would never go skydiving |
| Q36 | If I heard a noise in my house at night, I would go and investigate it |
| Q37 | I enjoy going on roller coasters |
| Q38 | I would happily swim with sharks |
| Q39 | I am scared of the dark |
| Q40 | I panic easily |
|  | DISGUST |
| Q41 | I would feel repulsed if I saw a cockroach |
| Q42 | I hate touching slimy things |
| Q43 | I would be disgusted to find mould on some food I was eating |
| Q44 | Smelling milk that has gone off makes me nauseous |
| Q45 | I avoid being near people who look ill |
| Q46 | I would not eat any food that had passed its sell-by date |
| Q47 | I would never use someone else's toothbrush |
| Q48 | I could not help a friend to clean their wound if it had pus in it |
| Q49 | Gory images make me feel faint |
| Q50 | I always keep my kitchen free from any germs |
|  | ATTRACT |
| Q51 | I would consider having a medical procedure to enhance my appearance |
| Q52 | I like to dress provocatively |
| Q53 | I spend a lot of time getting ready to go out on a date |
| Q54 | I exercise/work out so I look better to others |
| Q55 | I don't take great care of my appearance |
| Q56 | My friends would say I'm a flirt |
| Q57 | I would not be concerned if I started to look old |
| Q58 | I like reading articles about how to attract a mate |
| Q59 | I like to hang out where I might meet desirable partners |
| Q60 | I could seduce a stranger if I wanted to |
|  | LOVE |
| Q61 | I don’t tend to miss my partner much when they are away |
| Q62 | I am happiest when I am with a person I love |
| Q63 | I strive hard to make my partner secure in my love for them |
| Q64 | I'd rather spend time with my partner than do anything else |
| Q65 | Being dependable is the most important characteristic in a life partner |
| Q66 | I could potentially forgive my partner for cheating on me |
| Q67 | I see my partner as an extension of myself |
| Q68 | If I won some money I wouldn't feel obliged to spend it on my partner |
| Q69 | Finding your ideal life partner is the best thing that can happen to you |
| Q70 | I wouldn't mind being single for the rest of my life |
|  | NURTURE |
| Q71 | The smile of a child is one of the most beautiful things on the planet |
| Q72 | Being a parent is the most important role one can play in life |
| Q73 | Doing the little things that are needed to make sure a child is safe and secure give me satisfaction |
| Q74 | The mere thought that a child might be in danger gets me upset |
| Q75 | My career is more important to me than having children |
| Q76 | I would give my life to save my child's |
| Q77 | I would rather be a full time parent than have to work away from my children |
| Q78 | I really enjoy babysitting for my family members |
| Q79 | I (want to) work with children because I love to see them grow |
| Q80 | Members of my family come to me when they need help or support |
|  | HOARD |
| Q81 | I don’t take very good care of the things I own |
| Q82 | I have a reputation for being frugal |
| Q83 | I would rather mend something old than buy a new replacement |
| Q84 | It’s more important to save up for the future than to have a good time now |
| Q85 | I always like to keep plenty of spare items around just in case I need them |
| Q86 | I feel secure when I'm surrounded by stuff that might come in handy |
| Q87 | I'm always buying things that I don't really need |
| Q88 | I find it difficult to throw anything away |
| Q89 | I like to have complete sets of the things I collect |
| Q90 | If something is free I tend to take it even if I don't need it |
|  | CREATE |
| Q91 | I put off tidying up my surroundings until I really have to |
| Q92 | I enjoy making things from scratch |
| Q93 | I hate doing DIY at home |
| Q94 | I constantly make small improvements to the things I own |
| Q95 | I love planting seeds and watching them grow |
| Q96 | I'm not much use at making things |
| Q97 | I would like to build my own house |
| Q98 | I would enjoy the challenge of fending for myself on a desert island |
| Q99 | I appreciate good workmanship |
| Q100 | I like coming up with new inventions |
|  | AFFILIATE |
| Q101 | I don't have many friends |
| Q102 | I often would rather do something on my own than go out with a group of friends |
| Q103 | I spend a lot of time keeping in contact with my friends |
| Q104 | I can say I know a lot of people |
| Q105 | I'm not one to join clubs |
| Q106 | I prefer to work in a team |
| Q107 | I dislike parties and large social gatherings |
| Q108 | I am always worried about doing the right thing in a social situation |
| Q109 | I try hard to fit in with those around me |
| Q110 | I'm careful to imitate what my friends do or say |
|  | STATUS |
| Q111 | Much of what I do is designed to improve my social position |
| Q112 | I crave recognition for what I have achieved |
| Q113 | Holding a well-respected position in society is important to me |
| Q114 | I try to make myself appealing by being kind |
| Q115 | It is important to me to make a good impression when meeting people for the first time |
| Q116 | People in my social circle look up to me |
| Q117 | I feel I deserve extra courtesy because of who I am |
| Q118 | I enjoy showing off things that tell people I'm important |
| Q119 | I do not enjoy being in charge of other people |
| Q120 | Most people would say I'm not a competitive person |
|  | JUSTICE |
| Q121 | I would scold anyone who was inconsiderate to others |
| Q122 | I get angry when I see someone take advantage of others |
| Q123 | I would call out someone who tried to skip ahead in a queue |
| Q124 | I agree with the saying, "An eye for an eye, a tooth for a tooth" |
| Q125 | I would report someone to the police if they broke the law |
| Q126 | I would love a job that involves holding people to account for their actions |
| Q127 | I am not afraid to stand up for the right thing |
| Q128 | I would not be comfortable profiting from someone else's misfortune |
| Q129 | I have a deep interest in politics |
| Q130 | Criminals should be made to suffer for their crimes |
|  | CURIOSITY |
| Q131 | It would be a great thrill to discover something no one has ever known before |
| Q132 | I would rather get lost wandering around a new area than not have had the pleasure of exploration at all |
| Q133 | I get a lot of pleasure from discovering how things work |
| Q134 | I am fascinated by going to places I haven't visited before |
| Q135 | I read more fiction than non-fiction |
| Q136 | Studying the genetics of flies is a waste of time and money |
| Q137 | I always read to try and learn more about the world |
| Q138 | I am interested in everything |
| Q139 | I would rather get on with doing something than spend a long time learning about it |
| Q140 | I do not spend a lot of time thinking about why things are the way they are |
|  | PLAY |
| Q141 | Having fun in whatever I do is important to me |
| Q142 | I like to tease my friends |
| Q143 | I totally lose myself in the books I read |
| Q144 | Sports are a big part of my life |
| Q145 | I've always enjoyed play-acting |
| Q146 | I don't mind practicing something over and over if I get better at it |
| Q147 | I love to learn new skills |
| Q148 | I would never play a prank on someone |
| Q149 | Comedy is my favourite genre of film/television |
| Q150 | I enjoy contemplating new ideas |
